# Complete genome sequence of a high-risk *Escherichia coli* ST10 isolated from avian feces in Pakistan

**DOI:** 10.1128/mra.00769-24

**Published:** 2024-10-09

**Authors:** Jung Hun Lee, Abdul Rauf Tareen, Nam-Hoon Kim, Chanyeong Jeong, Byeonghyeon Kang, Gwangje Lee, Dae-Wi Kim, Rabaab Zahra, Sang Hee Lee

**Affiliations:** 1 National Leading Research Laboratory of Drug Resistance Proteomics, Department of Biological Sciences, Myongji University, Yongin, South Korea; 2 Department of Microbiology, Quaid-i-Azam University, Islamabad, Pakistan; 3 Department of Life Sciences, Jeonbuk National University, Jeonju, South Korea; University of Maryland School of Medicine, Baltimore, Maryland, USA

**Keywords:** *Escherichia coli*, avian feces, ST10, antibiotic resistance gene

## Abstract

We present the complete genome sequence of *Escherichia coli* strain PEC1009 isolated from avian feces in Pakistan. The strain belongs to sequence type (ST) 10, a high-risk clone. The strain possesses two plasmids, and various plasmid-borne antibiotic resistance genes and chromosome-borne virulence factor genes were identified in its genome.

## ANNOUNCEMENT

The presence of antibiotic resistance genes (ARGs) and virulence factors (VFs) in the opportunistic pathogen *Escherichia coli* represents a significant health issue worldwide, particularly in developing countries (1–3). Here, we report the isolation of an *E. coli* strain belonging to sequence type (ST) 10, originating from avian feces in Pakistan.

Droppings of fecal samples collected from Marghazar zoo, Islamabad, Pakistan (34.6678°N, 72.3431°E), were serially diluted in saline and spread on MacConkey agar plates. The plates were incubated at 37°C for 18–24 hours. Colonies displaying pink color were selectively isolated and subjected to biochemical assays, resulting in isolation of *E. coli* strain PEC1009. The strain was cultured overnight at 37°C in liquid lysogeny broth (LB) medium with 100 µg/mL ampicillin, and genomic DNA was extracted from the harvested cells using the Qiagen MagAttract HMW DNA kit (Qiagen, Germany). The Megaruptor3 (Diagenode, USA) was used to shear the genomic DNA, and small fragments were removed via AMpureXP bead purification (Beckman Coulter, USA). An SMRTbell library was constructed using the SMRTbell Express Template Preparation Kit (Pacific Biosciences, USA). The library was sequenced using Sequel Sequencing Kit v3.0 and SMRT Cell 1M v2, and the sequence data were obtained using the Sequel sequencing platform (Pacific Biosciences, USA). The sequencing data were assembled with SMRT Link based on the Microbial Assembly protocol (Pacific Biosciences, USA). Potential contamination in the genome assembly was assessed using the ContEst16S (4). A total of 424,164 reads were obtained, with the average subread length of 5,679 bp. Genome annotation was performed using the Prokaryotic Genome Annotation Pipeline (PGAP) v.6.7 (5). Circularization of the contigs was determined using the SMRT Link assembly protocol (Pacific Biosciences, USA). Circulator v.1.4.0 (Sanger institute) was employed to rotate them as the start position of *dnaA* or replication origin. Phylogroup assignment and Achtman scheme sequence typing were conducted using the ClermonTyping and the pubMLST, respectively (6, 7). Serotype, plasmid replicon sites, and virulence factors (VFs) were identified using the Abricate (v.1.0.0, https://github.com/tseemann/abricate) tool using ECOH (Nov. 2023), PlasmidFinder (Nov. 2023), and VFDB (Jun. 2024), respectively (Table 1) (8–10). ARGs were detected using the Resistance Gene Identifier (RGI) tool (v.6.0.3) (11). Default parameters were used for all software, unless otherwise specified.

**TABLE 1:** Virulence factors (VFs) in the chromosome of *E. coli* strain PEC1009

VF category					
ECP^*a*^	Enterobactin	LEE^*b*^ encoded T3SS	T2SS^*c*^	Yersiniabactin	Etc^*d*^
*yagV/ecpE, yagW/ecpD, yagX/ecpC, yagY/ecpB, yagZ/ecpA,* and *ykgK/ecpR*	*entA, entB, entC, entD, entE, entF, entS, fepA, fepB, fepC, fepD, fepG,* and *fes*	*espL1, espL4, espR1, espX4, espX5,* and *espY1*	*gspC, gspD, gspE, gspF, gspG, gspH, gspI, gspJ, gspK, gspL,* and *gspM*	*fyuA, irp1, irp2, ybtA, ybtE, ybtP, ybtQ, ybtS, ybtT, ybtU,* and *ybtX*	*aslA, astA, astA, csgB, csgD, csgF, csgG, fdeC,* and *ompA*

^*a*^ ECP, *Escherichia coli* common pilus.

^*b*^ LEE-encoded T3SS, locus of enterocyte effacement-encoded type III secretion system.

^*c*^ T2SS, type II secretion system.

^*d*^ Etc, Agf, AslA, curli fibers/thin aggregative fimbriae, EAST1, FedC, OmpA.

The complete genome of strain PEC1009 consists of three circular contigs: one chromosome (4,667,174 bp) and two plasmids, pPEC1009-1 (125,967 bp) and pPEC1009-2 (3,316 bp). The sequencing depth coverage was 435.9 x. The GC contents are 50.9% for the chromosome, 52.4% for pPEC1009-1, and 43.0% for pPEC1009-2. The strain belongs to ST10 and phylogroup A, with a serotype identified as H10. It carries FIB and Col plasmid replicon sites. Plasmid pPEC1009-1 carries eight ARGs (*bla*_TEM-1_, *bla*_CTX-M-15_, *aph(3'')-Ib*, *aph (6)-Id*, *qnrS1*, *sul2*, *tet(A*), and *dfrA14*). Various VF genes were also detected in the chromosome (Table 1).

## Data Availability

The genome is available at the NCBI BioSample SAMN42018128 within the BioProject PRJNA1127758. The genome sequences have been deposited in the NCBI GenBank database under accession numbers CP160468, CP160469, and CP160470. The raw sequence data are available under Sequence Read Archive accession number SRX25437700.
